# Supplementary material for: Entity Linking for real-time geolocation of natural disasters from social network posts
Source: PLoS One. 2024 Oct 7;19(10):e0307254. doi: 10.1371/journal.pone.0307254 (PMC11457996; doi:10.1371/journal.pone.0307254)
Supplement: S1 File — (PDF) [file pone.0307254.s001.pdf]

Supporting Information 1 - CAp 2017 label mappings

Cap 2017 label	Our labels
0	0
event	EVENT
geoloc	GEOLOC
facility	FACILITY
media	ORG
movie	OTHER
musicartist	PERSON
org	ORG
other	OTHER
product	OTHER
person	PERSON
sportsteam	ORG
transportline	TRANSPORT
tvshow	OTHER
