# Supplementary material for: Entity Linking for real-time geolocation of natural disasters from social network posts
Source: PLoS One. 2024 Oct 7;19(10):e0307254. doi: 10.1371/journal.pone.0307254 (PMC11457996; doi:10.1371/journal.pone.0307254)
Supplement: S2 File — (PDF) [file pone.0307254.s002.pdf]

Supporting Information 2 - HIP 2022 label mappings

HIP 2022 label	Our labels
0	0
pers.ind	PERSON
pers.ind.articleauthor	PERSON
pers.coll	ORG
org.adm	ORG
org.ent	ORG
org.ent.pressagency	ORG
loc.adm.town	GEOLOC
loc.adm.reg	GEOLOC
loc.adm.nat	GEOLOC
loc.adm.sup	GEOLOC
loc.phys.geo	GEOLOC
loc.phys.hydro	GEOLOC
loc.phys.astro	OTHER
loc.oro	TRANSPORT
loc.fac	FACILITY
loc.add.phys	FACILITY
loc.add.elec	OTHER
loc.unk	GEOLOC
time.date.abs	OTHER
prod.media	ORG
prod.doctr	OTHER
