# Supplementary material for: Entity Linking for real-time geolocation of natural disasters from social network posts
Source: PLoS One. 2024 Oct 7;19(10):e0307254. doi: 10.1371/journal.pone.0307254 (PMC11457996; doi:10.1371/journal.pone.0307254)
Supplement: S3 File — (PDF) [file pone.0307254.s003.pdf]

Supporting Information 3 - Twitter query for the Teil earthquake

```
("seisme" OR "seismes" OR "séisme" OR "séismes" OR
"tremblement de terre" OR "tremblements de terre" OR
"magnitude" OR "terre tremble") AND -"politique" AND -
"politiques"
lang:fr AND -is:retweet
```
