# Supplementary material for: Entity Linking for real-time geolocation of natural disasters from social network posts
Source: PLoS One. 2024 Oct 7;19(10):e0307254. doi: 10.1371/journal.pone.0307254 (PMC11457996; doi:10.1371/journal.pone.0307254)
Supplement: S4 File — (PDF) [file pone.0307254.s004.pdf]

Supporting Information 4 - Twitter query for the Alex storm

```
("inondation" OR "inondations" OR "inondé" OR "inondée" OR
"inondés" OR "inondées" OR "sous l’eau" OR "en crue" OR "la
crue" OR "crues" OR "décrue" OR "onde de crue" OR "sort de son
lit" OR "sorti de son lit" OR torrentiel OR "emport é par les
eaux" OR "emportée par les eaux" OR "emport és par les eaux"
OR "emportées par les eaux")
lang:fr AND -is:retweet
```
