# Supplementary material for: Entity Linking for real-time geolocation of natural disasters from social network posts
Source: PLoS One. 2024 Oct 7;19(10):e0307254. doi: 10.1371/journal.pone.0307254 (PMC11457996; doi:10.1371/journal.pone.0307254)
Supplement: Attachment — Submitted filename: PONE-D-23-07957-answers-to-reviewers.pdf [file pone.0307254.s005.pdf]

[PONE-D-23-07957] - Revision of the manuscript

We would to thank the two anonymous reviewers and the editor for their deep and useful comments. It really helped us to prepare a better version of the manuscript. It took us quite some time to revise the paper because we had to rerun evaluations. We hope that the paper is clearer now. Our answers to all comments can be found in red below.

When submitting your revision, we need you to address these additional requirements.

1. Please ensure that your manuscript meets PLOS ONE's style requirements, including those for file naming. The PLOS ONE style templates can be found at https://journals.plos.org/plosone/s/file?id=wjVg/PLOSOne_formatting_sample_main_body.pdf and https://journals.plos.org/plosone/s/file?id=ba62/PLOSOne_formatting_sample_title_authors_affiliations.pdf

2. In your Methods section, please include additional information about your dataset and ensure that you have included a statement specifying whether the collection and analysis method complied with the terms and conditions for the source of the data.
   Our dataset is now available on the ZENODO platform: https://doi.org/10.5281/zenodo.7767294. A reference to this download link has been added, as well as a sentence indicating compliance with the terms of use of the source data, regarding the conditions defined for the Academic Research access to the Twitter API.

3. Please note that PLOS ONE has specific guidelines on code sharing for submissions in which author-generated code underpins the findings in the manuscript. In these cases, all author-generated code must be made available without restrictions upon publication of the work. Please review our guidelines at https://journals.plos.org/plosone/s/materials-and-software-sharing#loc-sharing-code and ensure that your code is shared in a way that follows best practice and facilitates reproducibility and reuse.

Our code is deposited on a Gitlab repository whose link is shared in the paper. For now, reviewers can access the code and the weights of the model on https://figshare.com/s/3d5faa5258d1346dbe01.

4. Thank you for stating the following in the Acknowledgments Section of your manuscript:
   "The work presented in this article was carried out with funding from the Agence Nationale de la Recherche (ANR) within the framework of the CARNOT institutes, as well as within the the R´eSoCIO project co-funded by ANR under the grant ANR-20-CE39-001. Opinions expressed in this paper solely reflect the authors' view; the ANR is not responsible for any use that may be made of information it contains. The authors would like to thank F. Boulahya, Y. Retout, C. Mato, A. Montarnal, B.Farah and F. Smai for their help in annotating data, as well as the MAIF Foundation for its support to the design and development of the SURICATE-Nat platform. We also thank BCSF for giving us access to its French macroseismic data distribution webservice, and L. Bernede from M´et´eo France for providing us with the rainfall data recorded during Alex storm."
   We note that you have provided funding information that is not currently declared in your Funding Statement. However, funding information should not appear in the Acknowledgments section or other areas of your manuscript. We will only publish funding information present in the Funding Statement section of the online submission form.
   Please remove any funding-related text from the manuscript and let us know how you would like to update your Funding Statement. Currently, your Funding Statement reads as follows:
   "The work presented in this article was carried out with funding from the French Research Agency (ANR - https://anr.fr/en/) within the framework of the CARNOT institutes (Gaëtan Caillault), as well as within the the RéSoCIO project co-funded by ANR under the grant ANR-20-CE39-001 (Samuel Auclair & Cécile Gracianne). Opinions expressed in this paper solely reflect the authors' view; the ANR is not responsible for any use that may be made of information it contains.
   The funders had no role in study design, data collection and analysis, decision to publish, or preparation of the manuscript."
   Please include your amended statements within your cover letter; we will change the online submission form on your behalf.
   We have removed the mention of funding sources in the acknowledgements section, and confirm that the information given in our amended statements are correct.
5. In your Data Availability statement, you have not specified where the minimal data set underlying the results described in your manuscript can be found. PLOS defines a study's minimal data set as the underlying data used to reach the conclusions drawn in the manuscript and any additional data required to replicate the reported study findings in their entirety. All PLOS journals require that the minimal data set be made fully available. For more information about our data policy, please

see http://journals.plos.org/plosone/s/data-availability.
"Upon re-submitting your revised manuscript, please upload your study's minimal underlying data set as either Supporting Information files or to a stable, public repository and include the relevant URLs, DOIs, or accession numbers within your revised cover letter. For a list of acceptable repositories, please see http://journals.plos.org/plosone/s/data-availability#loc-recommended-repositories. Any potentially identifying patient information must be fully anonymized.
Important: If there are ethical or legal restrictions to sharing your data publicly, please explain these restrictions in detail. Please see our guidelines for more information on what we consider unacceptable restrictions to publicly sharing data: http://journals.plos.org/plosone/s/data-availability#loc-unacceptable-data-access-restrictions. Note that it is not acceptable for the authors to be the sole named individuals responsible for ensuring data access.
We will update your Data Availability statement to reflect the information you provide in your cover letter.
The dataset presented in the study is available via the ZENODO platform (https://doi.org/10.5281/zenodo.7767294), and reviewers can access the code and the weights of the model on https://figshare.com/s/3d5faa5258d1346dbe01.

6. We note that Figure 4 and 6 in your submission contain [map/satellite] images which may be copyrighted. All PLOS content is published under the Creative Commons Attribution License (CC BY 4.0), which means that the manuscript, images, and Supporting Information files will be freely available online, and any third party is permitted to access, download, copy, distribute, and use these materials in any way, even commercially, with proper attribution. For these reasons, we cannot publish previously copyrighted maps or satellite images created using proprietary data, such as Google software (Google Maps, Street View, and Earth). For more information, see our copyright guidelines: http://journals.plos.org/plosone/s/licenses-and-copyright.

We require you to either (a) present written permission from the copyright holder to publish these figures specifically under the CC BY 4.0 license, or (b) remove the figures from your submission:

a. You may seek permission from the original copyright holder of Figure 4 nd 6 to publish the content specifically under the CC BY 4.0 license.

We recommend that you contact the original copyright holder with the Content Permission Form (http://journals.plos.org/plosone/s/file?id=7c09/content-permission-form.pdf) and the following text:

"I request permission for the open-access journal PLOS ONE to publish XXX under the Creative Commons Attribution License (CCAL) CC BY 4.0 (http://creativecommons.org/licenses/by/4.0/). Please be aware that this license allows unrestricted use and distribution, even commercially, by third parties. Please reply and provide explicit written permission to publish XXX under a CC BY license and complete the attached form."

Please upload the completed Content Permission Form or other proof of granted permissions as an "Other" file with your submission.

In the figure caption of the copyrighted figure, please include the following text: "Reprinted from [ref] under a CC BY license, with permission from [name of publisher], original copyright [original copyright year]."

b. If you are unable to obtain permission from the original copyright holder to publish these figures under the CC BY 4.0 license or if the copyright holder's requirements are incompatible with the CC BY 4.0 license, please either i) remove the figure or ii) supply a replacement figure that complies with the CC BY 4.0 license. Please check copyright information on all replacement figures and update the figure caption with source information. If applicable, please specify in the figure caption text when a figure is similar but not identical to the original image and is therefore for illustrative purposes only.
The following resources for replacing copyrighted map figures may be helpful:

USGS National Map Viewer (public domain): http://viewer.nationalmap.gov/viewer/
The Gateway to Astronaut Photography of Earth (public domain): http://eol.jsc.nasa.gov/sseop/clickmap/
Maps at the CIA (public domain): https://www.cia.gov/library/publications/the-world-factbook/index.html and https://www.cia.gov/library/publications/cia-maps-publications/index.html
NASA Earth Observatory (public domain): http://earthobservatory.nasa.gov/
Landsat: http://landsat.visibleearth.nasa.gov/
USGS EROS (Earth Resources Observatory and Science (EROS) Center) (public domain): http://eros.usgs.gov/#
Natural Earth (public domain): http://www.naturalearthdata.com/
Figures 4 and 6 use only the outline of French departments provided by OpenStreetMap contributors under ODbL license (https://www.data.gouv.fr/fr/datasets/contours-des-departements-francais-issus-d-openstreetmap/#/community-reuses). Reference to the data source has been added to the legend of each figure.

6. We note you have included a table to which you do not refer in the text of your manuscript. Please ensure that you refer to Table 5 in your text; if

accepted, production will need this reference to link the reader to the Table.

This was a mistake from our part. We removed the table as it was not related to the current text.

Additional Editor Comments:
You are required to update your manuscript based on the comments of the reviewers .

[Note: HTML markup is below. Please do not edit.]

Reviewers' comments:

Reviewer's Responses to Questions
Comments to the Author

1. Is the manuscript technically sound, and do the data support the conclusions?

The manuscript must describe a technically sound piece of scientific research with data that supports the conclusions. Experiments must have been conducted rigorously, with appropriate controls, replication, and sample sizes. The conclusions must be drawn appropriately based on the data presented.
Reviewer #1: Yes
Reviewer #2: Partly

---

2. Has the statistical analysis been performed appropriately and rigorously?
   Reviewer #1: Yes
   Reviewer #2: No

---

3. Have the authors made all data underlying the findings in their manuscript fully available?

The PLOS Data policy requires authors to make all data underlying the findings described in their manuscript fully available without restriction, with rare exception (please refer to the Data Availability Statement in the manuscript PDF file). The data should be provided as part of the manuscript or its supporting information, or deposited to a public repository. For example, in addition to summary statistics, the data points behind means, medians and variance measures should be available. If there are restrictions on publicly sharing data—

e.g. participant privacy or use of data from a third party—those must be specified.
Reviewer #1: No
Reviewer #2: No

---

4. Is the manuscript presented in an intelligible fashion and written in standard English?

PLOS ONE does not copyedit accepted manuscripts, so the language in submitted articles must be clear, correct, and unambiguous. Any typographical or grammatical errors should be corrected at revision, so please note any specific errors here.
Reviewer #1: Yes
Reviewer #2: Yes

---

5. Review Comments to the Author

Please use the space provided to explain your answers to the questions above. You may also include additional comments for the author, including concerns about dual publication, research ethics, or publication ethics. (Please upload your review as an attachment if it exceeds 20,000 characters)

Reviewer 1

This paper discusses the challenges of using social networks to develop situational awareness during natural disasters and proposes a method for detecting and geolocating French messages on Twitter to build maps in real-time. The authors demonstrate that their system performs as well as state-of-the-art systems and can contribute to automatic social network analysis for crisis managers. In my opinion, this work turns out to be technically valid even if it shows limitations as specified below:

- Novelty aspects: explain the novelty aspects of the paper better, also through concrete examples. The paper seems to be not very innovative, i.e. the application of known techniques adapted to the French context.

On the contrary, we do think that the adaptation to the French language is just one of the contributions of this work. We changed the end of the introduction to better reflect what we believe are the main contributions of this research.

- Related work (1): For each manuscript cited the main differences with the proposed approach must be highlighted. A comparative table could help the readers to understand the differences among the different works present in the literature and the strengths of this work.

We do think that there is no solution for our problem in the literature. The first reason is obvious: we need a model able to process French tweets to be used by French crisis managers. But beyond this language problem, we need precise and unambiguous locations inferred from the tweets. We tried to better highlight the differences of our approach compared to the literature in the "Related Work" section but also elsewhere in the paper. We did not include a comparative table to limit as much as possible the length of the paper.

- Related work (2): The paper "Using Social Media for Sub-Event Detection during Disasters" it turns out to be a related work very close to the one proposed. In particular, it proposes a technique for identifying the sub-events that occur after a disaster. How is your work different?

Thank you for pointing this recent paper we did not know of. We obviously included it in the citations and we discussed the differences with our work in the modified Section 2.1.

- Dataset and code: To allow the reproducibility of the experiments it is necessary to publish the datasets on a public repository and share the link in the paper. Also, the method code should be made public to allow experiments to be reproduced and the results obtained to be validated.

Data and code will be publicly available as soon as the paper is definitively accepted through the zenodo plateform and a github repository. Both URLs will be added to the manuscript.

- Experiments: Since the authors of this paper have a dataset with labels, why didn't you carry out quantitative analyses on the accuracy of your method (F1-score) in detecting events?

Our model was trained on the complete annotated dataset, this is why we did not provide any quantitative result. We annotated a new set of tweets, and we provide the quantitative analysis of the model based on this test dataset.

Reviewer 2

In this paper, the task of detecting and geolocating information in French language is addressed. The contributions are a data set and an entity-linking

pipeline. Due to the following reasons, I would like to recommend that the paper needs significant revisions before publishing:

- the proposed models need more detailed descriptions

We tried to improve the description of our model and its variants.

- the narrative of the paper needs to be enhanced to ensure that the reader can clearly see “the plot”

We improved the introduction and the literature review to help the reader to see “the plot”. We hope that the narrative is now clearer.

- the task is not well described and changes from EL (place mentions in texts) to geolocation of natural disasters - the latter is definitely not in scope of the involved methods as single tweets are processed (geo-located)

Our task is neither the pure EL problem nor the geo-location of natural disasters. Our long term goal is to provide useful information to crisis managers and rescuers with tweets containing geo-located information about the disaster. EL is the method that we feel is the most suited for our task, while the geo-location of natural disasters is just an interested information derived from the geo-location of the tweet contents. We tried to clarify the task in the introduction.

- the training data itself is partially generated from ML models, of which the actual performance is unclear - this needs to be further investigated

You are right. We hope that the new evaluation section gives better insights on the performances of the model.

- more quantitative experiments related to the proposed models are missing but definitely required: how well is the NER-step working? how do errors propagate? what is the effect of the cross-encoder?

We ran new evaluations to get a quantitative assessment of the model accuracy (see Section 5 of the revised manuscript).

- an interpretation of the scores in table 6 is missing: what are reasons for the poor F1-scores?

Our model was trained on a different task, which explains the poor values in the table. As we now have a quantitative evaluation on the task the model is designed for, we removed this whole part of the paper.

- while also highlighted in the manuscript title, the real-time aspect is not addressed at all

You are right, the context of a real-time geo-location is not really discussed in the paper, though it is one of the constraints that guided our decisions when designing this model (other approaches in the literature can have more accurate predictions but do not provide real-time predictions). We tried to add this criteria in our discussion of the literature.

- Why are entities involved, which will actually have not coordinate (e.g. person)?

This was due to a request by our users, the crisis managers, who are interested in as much information as possible. But we do not have enough instances of those annotations in our tweets, so the results are not significant. We decided to remove all the superfluous annotations in the revised manuscript.

More detailed comments:

Abstract

“show that despite these additional constraints” --> not sure to which constraints is referred here?

We changed the wording of the sentence to show that the constraints are cited in the sentence just before.

Introduction

32/33 “one of the main issues of these automatic analyses is the ability to correctly place the information extracted in a map” - might be, but not the first one - how about overload reduction?

The effect of information overload is well known in crisis management, and can be exacerbated by monitoring social networks, which may provide enormous amount of raw data that crisis managers find difficult to manage. Following numerous discussions with these crisis managers, it is clear that their problem is to be able to easily extract relevant information from posts, and to geolocate them in order to represent them on a map: it is this process of extracting information and representing it spatially that ultimately helps to reduce this information overload.

While reading the first two chapters, a question comes up: what is the actual motivation of entity linking here? Why do the authors expect a benefit for the

task (hypothesis)? It may be clear or the reader might have an idea, but reading the author's perspective would give more insights here.

Motivations to rely on Entity Linking are multifold: avoid ambiguous matchings with gazetteers, gain in precision, allow the inference of places not existing during training phase... These reasons have been added at the beginning of the related works section.

61: "we propose a pipeline to automatically geolocate natural disasters from tweets" --> this sentence suggests that events will be detected, but the task is related to single tweets, when I get it right. Hence, geolocating "natural disasters" might not be the appropriate term here.

The wording has been changed in the article.

Would actually be good to also read from the obtained results / performance abilities of the proposed method in the introduction

We added a brief outlook of the results in the description of the contributions.

Task description and related works

I like that we can find a description of the task first. It may be good to provide a list of examples to explicitly (1) show the different types of toponyms, the authors are actually addressing, and (2) the target types of place description (points, polygons,...?). Later (2.2), we can find

The task "mentioned location prediction" is also known as geoparsing.

Thank you for your suggestion, we added this information in the paper.

"but we will focus more on methods addressing a similar crisis management context" I can understand this approach, but sometimes it is worth doing a review independently from a domain - it may turn out that there are approaches around that are not well known in disaster management but worth to test? Just a thought...

You are completely right and it is exactly what we did in this project, as our ideas derive from readings in domains completely different from disaster management. This comment was only meant to describe the papers reviewed in the manuscript. There is no space for a complete review of methods to geolocate tweets, and there are even several review papers that were published recently. We change our phrasing to clarify this sentence.

In line with reference 26, this one might also be of interest: https://ieeexplore.ieee.org/abstract/document/9711571

Thank you for your suggestion. Indeed, we cited the first version of GazPNE, but we did not know this more recent publication. We included it in the revised manuscript.

In addition to the mentioned approaches in the related work, it would be good to also read about the open issues / drawbacks they have - at least those that are addressed in this paper.

After each mentioned approach from the literature, we tried to explain its drawbacks, at least the ones that we think we address with our model.

While reading 2.2, I am wondering why the description of the EL task is not part of the introduction of the chapter (where the task is described)? This would give a comprehensive overview of all involved sub-tasks that are addressed in this work followed by related work. In turn, chapter 2.2 focuses a lot on the task and only a few related works are mentioned. I would recommend to re-structure this chapter.

We might be wrong but we feel that section 2.1 would be odd if the Entity Linking task was presented first, as most approaches to our task do not use EL. As we restructured Section 2.1, we decided to keep the initial structure for EL and Section 2.2. If you still feel the EL task does not belong to 2.2, we would be happy to adopt your proposed order of presentation.

134: The meaning of BIO could be explained.

We added the following explanations "…where token labels are usually following the BIO scheme, where starts of mentions are labelled with B-X labels (X being the type of the entity), in-mention tokens with I-X labels and other tokens with O."

156: dated --> outdated?

The correction has been made.

180: "Detecting and geolocating natural disasters" --> this suggests that events are detected. However, this is not in line with the task description, which works on a document-level

The wording has been changed in the article.

3.1: Here, the first part of the model is described. For better understandability, I would favor to get a first overarching overview of the proposed method followed by the detailed descriptions.

We added a short paragraph briefly giving an overview of the complete framework (bi-encoder + cross-encoder).

Figure 1: In its current form, the figure does not contain all relevant information. For instance, what do the green vectors/matrices contain/represent? How is the final embedding actually is computed?

A quick introduction has been added to give an overview of the model. Plus, more details have been added to explain how each part of the model are connected.

211: "At inference time, entity embeddings can be pre-computed,": This statement is a bit confusing, as the embeddings for the entities are already pre-computed (i.e., at inference-time, only the mention embedding has to be computed)? Which metric is actually used to compare embedding vectors?

More details are given in the revised manuscript, and the explanation should be clearer.

217ff: "Then, we propose to rely on the first token, instead of the CLS token, of an entity mention to produce mention embeddings, thus allowing to embed all the entity mentions of a document at the same time." --> It would then be required to explain, how multiple mentions - especially the expected varying number - is handled

More details are given in the revised manuscript, and the explanation should be clearer.

218: "we propose to rely on the first token": why not B and I considered (as some mentions might consist of more than 1 or 2 tokens)? OK, addressed in line 225...

Each token classified as B (by the NER classifier) are selected to be mention embeddings. So, each token embeddings (outputted by the language model) are given to NER classifier. Then, all word embedding classified as B are given to the last feedforward neural network to build the final mention embedding.

3.2 Cross-Encoder: The description is not quite detailed and could be enhanced. How does it eventually help to mitigate the aforementioned potential errors?

We actually did not really put much effort in the design of the cross-encoder. It is literraly the same approach that have been used in "Zero-shot entity linking by reading entity description", so we don't have much to say about this part of the

model, except that it helps us to filter out "bad candidates" that may have been selected by the bi-encoder.

288: “the best performances are obtained with”: this statement seems to be quite holistic - I would rather say that this is true according to the conducted experiments in the mentioned paper?

This part has been changed in the revised manuscript.
324: “beforehand, The” -> the

Thank you for your comment, it is corrected in the revised manuscript.

342ff: When I see the list of annotation labels considered, I am a bit confused. Taking into account types that do not describe places differs from the task?

This is true that PERSON and ORG classes are not required in the current state of this work. We choose to keep them since these labels are almost always given in NER datasets. While we could have got rid of them in this specific task, we chose no to because our work might be useful in more general contexts and because those labels might convey geographical knowledge when they refer to local representatives/associations (for instance). We tried to explain this better in the revised manuscript.

360: "We then applied this classifier to classify all the French Wikipedia entities ": This tends to be critical, as the labels will potentially not have the quality of manual annotations. It would be required to read about test results using unseen test data.

Indeed, you are right. Especially considering the very low amount of labelled data we have. We evaluated our model on 10% of the annotated dataset and found out the model was able to reach 0.75 fscore. While it's far from soat models, this is the best we can do with the quantity of data we had. We added more details in the updated paper.

385ff: These two sentences are representative for the writing style of the whole article, where the order of information presented appears to be a bit confusing. I just had a look the the paper “Entity Linking in 100 Languages” - the writing style is very crisp, clear and structured. I would recommend to revise the manuscript to achieve that readers can follow a bit easier.

We are really sorry about the quality of writing, and we did revise the language entirely. We hope that the style is now better, and that it is at least not confusing.

4.3: It is not clear, how the data is used when the EL-related part is missing?

The model cannot be trained on the EL task with the Cap2017 dataset. However, the dataset can be used to (1) train the language model on tweets so it is not confused at inference, and (2) train the NER classifier on tweets. It should help the model to correctly extract mention spans from tweets, which is the first step to a good EL model. Then, we rely on other datasets to train the model on the linking step.

400: "it then appears to be extremely valuable to help the model build coherent representations for tweets" --> this statement needs to be confirmed.

True, we never prove that it is indeed valuable. We replaced «it is valuable» with «it should be valuable»
406: "geolocate natural disasters from social networks": well, event if this is the application, it is still about place mentions, right? I would say that it of course depends on the involved document types - if Twitter is the target platform, a model needs to be trained with data from Twitter. But it might not be necessary to use disaster tweets to detect and link the place names. Might be a good idea for an experiment?

As explained in the introduction, we already have a system filtering tweets related to natural disasters. The experiment presented in the manuscript only uses the tweets that were filtered by this system, based on key words. But obviously, the task is still to geo-locate mentions in tweets.

Table 5 seems not to be mentioned or referenced in the text.

This table was an artifact of our own editions of the paper, we are really sorry about that. Anyway, as the evaluation sections were largely modified, the table does not appear in the paper anymore.

Table 6: EL performance is shown here. It would also be interesting to see, how well the mentions actually are detected as this would directly influence the linking. A (maybe stupid) question related to this: what would happen, if we simply take the NER results search the entities with these keywords (maybe allowing for some letter permutations)?

This part was completely removed from the manuscript, replaced by our new quantitative evaluation.

473: "given in Appendix 7 and 7"–> 7 appears twice

This was corrected in the revised manuscript, thank you.

493: "Since our model detects non-geographical entities too": this rises the question, why the other types are then actually still included? Doesn't this make the task more complex for the models?

You are right, there is a chance that trying to predict more than geo-locations makes the model less performant on this geo-location task. We did not conduct any ablation study due to time constraints.

Table 7: “The number between parenthesis indicates the number of tweets, mentions or entities which have been localized inside the area impacted by the earthquake/storm.”: as there are around 250 distinct entities found, it would be of interest (and feasible in terms of effort) to know the quality here (i.e., false positive rates or other metrics?).

As mentioned throughout our responses, we did conduct a quantitative evaluation of the model, but with different tweets than the ones used in the qualitative evaluation.

498: EMSC should be explained

The meaning of EMSC (European-Mediterranean Seismological Centre) has been clarified in the text.

548: “While this last analysis does not prove that our model predictions are correct”… this was exactly what I was thinking here: I would at first be interested in how well the proposed models actually perform - this is necessary. Another aspect: it is well known, that keyword-based filtering of tweets comes at the cost of a bad precision. How is ensured, that non-related tweets are not used in this analysis?

Our study is resolutely positioned as a contribution aimed at providing effective assistance to crisis management practitioners, and is therefore placed from their point of view with the priority of making sense of the situation rather than of the tweets themselves. Thus, while the previous sections were aimed precisely at assessing the performance of our model, we felt it important to look at the correlation between “predicted” elements in the digital sphere and the reality of the effects of natural disasters, something that is very rarely done in the literature.
Furthermore, it is clear that the keyword search is very restrictive, notably through the non-capture of relevant tweets (false-negatives), but this is a constraint imposed by Twitter. As for the irrelevant tweets collected here (i.e. false-positives), their effect remains very limited for the entity linking and geolocation task considered here. From an operational point of view, we are also working on the development of supervised classifiers to filter out irrelevant tweets for crisis managers.

6.2: As the title is “Alex storm”, it is a bit surprising/unexpected to read about floods in 6.2.2?

As mentioned in 6.2.1, Storm Alex resulted in very heavy rainfall: a large proportion of storm-related losses were due to flooding. Further details have been provided to clarify this point, in 6.2.1 and 6.2.2.

598 (Discussion): “Our model seems to be able to capture coherent representations of real natural disasters.” --> very vague - has to be underpinned with quantitative experiments

Thank you for this very pertinent comment. A quantitative analysis has therefore been added, in the form of a table (#8) and a discussion paragraph.

638: “Without being able to assess in detail the capacity of the model to detect all the geo-locatable features with precision”: why not taking the usual approach of random samples?

At the time of the first submission, we had used all the annotated tweets to train the model. We were aiming the annotation of test dataset of geo-located tweets, but due to time constraints, we never did. During this last months, we performed the additional annotation for a test dataset, and we performed the evaluation of our model with this test dataset, as mentioned earlier in our responses. So the cited sentence here has been removed.

640: “the model is able to capture the overall footprint of earthquakes and flash floods”: the proposed model can actually do EL and since the linked entities have geo-coordinates, they can be shown in a map. The fact that a footprint is visible, is related to the data and the users that report on an event. however, it is not clear, if the data contains many eyewitness-reports or (as commonly observed) sympathy/support messages. as the tweets are basically identified based on keywords, it is likely, that there are many false positives contained. this needs to be investigated based on quantitative and qualitative experiments.

This is absolutely right, and we’re well aware of it. From an application perspective, it will indeed be critical to be able to filter tweets according to their relevance, as well as the nature of the information they contain. In the context of the present contribution, which focuses on the ability to geolocate information correctly, this issue is not central, however, and we have therefore decided not to study it in detail. However, a comment has been added to the text to make this point more explicit for the reader.

650: “Furthermore, external expert knowledge, such as maps,…” this is actually a crucial point. no emergency response manager will solely decide based on a twitter system. they in fact already have well-established routines and have local knowledge so that we need to identify the information gaps

The point here is to indicate that this external knowledge can help the alorithms in the disambiguation process. On the other hand, it’s true that data from social media is just one more piece of information among many available to crisis managers.
