# Supplementary material for: Entity Linking for real-time geolocation of natural disasters from social network posts
Source: PLoS One. 2024 Oct 7;19(10):e0307254. doi: 10.1371/journal.pone.0307254 (PMC11457996; doi:10.1371/journal.pone.0307254)
Supplement: Attachment — Submitted filename: PLOSONE-Entity-Linking-forreal-time.pdf [file pone.0307254.s006.pdf]

Review for: Entity Linking for real-time geolocation of natural disasters from social network posts

This manuscript presents an entity-linking pipeline for geolocating French Tweets posted during natural disasters. Furthermore, the manuscript introduces an annotated entity-linking data set consisting of French Wikipedia corpus and French tweets collected during major disasters. I would recommend accepting this paper after clarifying a few points (elaborated below).

Strengths:

- The qualitative evaluation is good, demonstrating the utility of the proposed model for disaster response.

- The paper is well-written. The organization of the paper could be improved, but it remains easy to follow.

- The creation of an annotated data set that can be used for disaster response Named Entity Recognition and Entity Linking tasks in other languages is a valuable contribution, given that the mainstream research in this area is focusing on English data.

Concerns:

- The authors should clarify whether the proposed entity-linking pipeline presented in this manuscript presents a novel contribution. Are there similar entity-linking models that have been proposed to address this specific issue? Is the proposed architecture unique? A comparison with previous works is recommended.

- The cross-encoder component in the proposed pipeline is not clear. Clarifications are needed on whether this cross-encoder was trained separately or on the output of the bi-encoder. It will be helpful to add a figure illustrating all the pipeline's components and how they are linked to each other's.

- The categories of 'RISKNAT' and 'DAMAGES' (page 11) are unclear. Are they location-related categories? How do they differ from the 'GEOLOC' category? The authors provided a few examples of the 'RISKNAT' category in page 17 “information describing the phenomenon” such as “heavy rain” and “strong gusts of wind”, which do not seem to be location related. It would be beneficial if the authors explain more what these two categories are referring to in page 11.

- In section 5.2 (Results), the authors stated "Recently, a comparison of several NER models showed that state-of-the-art F1 scores are smaller than 75% [64], meaning that the results of the Bi-Encoder are excellent". It is not clear whether the F1 scores mentioned here are those trained and tested on French data. If it is the case, I think the F1 score obtained on French and English data are not directly comparable. There might be some variations in the way locations are mentioned in these two languages. Even though the scope of this paper is French tweets, I would suggest training and testing the NER component of the proposed pipeline on an existing English Benchmark (e.g., the one proposed in "Suwaileh R, Elsayed T, Imran M, Sajjad H. When a disaster happens, we are ready: Location Mention Recognition from crisis tweets. International Journal of Disaster Risk Reduction. 2022; p. 103107") and compare its performance to the state-of-the-art NER models to support the above conclusion.
