# Supplementary material for: Entity Linking for real-time geolocation of natural disasters from social network posts
Source: PLoS One. 2024 Oct 7;19(10):e0307254. doi: 10.1371/journal.pone.0307254 (PMC11457996; doi:10.1371/journal.pone.0307254)
Supplement: Attachment — Submitted filename: Reply to reviewers.pdf [file pone.0307254.s007.pdf]

We would to thank the reviewers and the editor for their useful additional comments. We took their recommendations into account and answered their questions. Our answers to all comments can be found in red below.

Reviewer #3: In general, the paper looks well-worked-upon and useful; I need to especially praise the authors for their willing to improve a concrete practice, rather than elaborate on a method that will never be actually used in real life.

I am not questioning the general approach; neither I am against the method in its major elements (design, pipeline, evaluation etc.). I appreciate the work with the datasets; they include nearly all available relevant data, as well as the new training dataset created by the authors. But I have several reservations that, to my viewpoint, need to be addressed, to make the paper less 'technical' and a bit closer to real life, including the practical disaster fighting by both professionals and ordinary people. These include conceptual issues and issues of methodology.

CONCEPTUAL ISSUES

1. The information on disasters is usually gathered not from social media (which may be highly misleading, as no fact-checking is done), but by special taskforces. Thus, why use your tool instead of using the fact-checked evidence collected by professionals? And what to do when the data from the former contradict the latter? What would your advice be? I think this issue needs to be mentioned in the paper, as stating unequivocally that such a tool only helps may, too, be misleading. We know from many examples, even before Twitter, e.g. from fires in Greece and other European countries that people tended to listen to media that reported on fires, not the firemen, and came to wrong places for help. Social media are an even more distorted mirrors of events; they may intensify, distort, misrepresent, and diminish the scale of the event. Thus, it needs to be clarified in the paper how exactly the tool is used and what for, knowing that social media may mislead the viewer, and how the info is doublechecked; this will explain why and how you wish to enhance its work.

Reply: Thank you for this very interesting comment. First of all, it is very true to point out that monitoring on social networks in support of crisis management is often carried out manually by teams of volunteers. Our aim is therefore not to replace these teams, but rather to support them by developing automatic processing tools allowing them to increase efficiency. The value of human analysis remains very important, especially to verify the extracted information. A paragraph has been added to this effect in the introduction.

2. However, even if your tool will represent the social media reality 100%, there is no guarantee that it corresponds 100% to what is happening on the ground. This, in its turn, means that the uses of the tool need to be limited to, say, disaster alerts – or, on the opposite, the tool needs to be ML-enhanced to be able to go beyond naïve search for locations and keywords but get trained to detect markers of both disasters and locations beyond particular names/keywords, in order to critically improve its quality.

Reply: We fully agree with the reviewer that social networks should not be used alone to build situational awareness, at the risk of getting a distorted vision of the reality. This is addressed in the introduction, which points out that social networks must be considered "in addition to traditional actionable channels". Furthermore, we believe that extracting geolocation information is a way of complementing naïve search with advanced analysis, enabling for example the detection of spatio-

temporal anomalies. However, how this information extracted from messages can be used to infer higher-level actionable intelligence is outside the scope of this article.

METHODS AND RESULTS

3. The authors are right in saying that there are too many papers on Twitter geolocation; but I am not sure they are right in disregarding all those that tackle issues beyond natiral disaster detection. This is especially wrong in terms of methodology: Many papers that do not focus on monitoring disasters still provide valuable improvements for the detection method itself. A short review may be found in Blekanov et al. (2022) – see https://dl.acm.org/doi/abs/10.1007/978-3-031-22131-6_2. As an example of what is omitted by the authors, this paper itself might also be of help, as it compares three approaches to enhance geolocation of Twitter users, including via ML-based analysis of their text corpora, and reports improving user geolocation. I am not sure how it will work on the community/local level, but it worked well on national level, as the authors claim.

Reply: Works on the geolocation of tweets may indeed be of methodological interest for our purpose, even if they do not deal directly with natural disasters. We were primarily interested in analyses of tweets sent during natural disasters, as these have the triple advantage of presenting not only the geolocation approaches implemented, but also the datasets used and the evaluation methodologies adopted for this particular use case. This enabled us to build a training corpus adapted to the texts to be processed, and to propose evaluation measures appropriate to the level of detail of the mentions of named spatial entities to be localized.

The approach proposed in the article referred to remains interesting in our case: thank you for bringing this to our attention. This work is based on the same steps as the ones we already present in the related woks section: here, the aim is to use a deep neural network-based NER model to recognize places mentioned in tweets (provided by the Spacy library), and then to associate the recognized mentions with coordinates using a geocoding tool (OSM Nominatim). This second step involves a linking operation between each extracted mention of a named place and a knowledge base of reference locations. A graph of users' interactions is added to improve the named place disambiguation process. This is in line with approaches presented in our state of the art, such as that of (Jurgens, 2013), which also uses the relationship graph of twitter users.

The approach we propose differs from that presented in this article in that it performs these two successive tasks in a single step. This has the advantage of allowing the textual context of spatial named entities, rich in spatial relationships with neighboring named places, to be taken into account during the linking stage, thus facilitating the disambiguation of candidate places. We have therefore added a reference in the article to clarify our positioning in relation to this kind of approaches.

4. In connection with (4): Analysing individual tweets may be productive for 'red alerts', but may remain endlessly non-productive for geolocation detection, however good annotation, model training, and fine-tuning may be. As a 'future work' prospect, I would still mention analysis of tweet pools. You tell that parsing the history of the network is unfeasible when you discuss such options, and claim you do not use prior knowledge in your model; but this is not about parsing the whole network but only the tweets of a particular account, of which the tweets would allow for quicker recognition of the residence of a given user who posted the info on a disaster. The same goes from the friendship network, but here the data may be more misleading (e.g., if one would detect my location based on my Facebook feed, they would be wrong by 4,400 km).

Reply: We purposely chose not to analyze individuals for three main reasons. First, we aim to geolocate the events mentioned in the social media post. As you said, user data is not fully trustable, but even if it were, the user's home location can be misleading for many reasons as people often refers to event far from where they live, or they can be away for vacation…

The second reason is that we did not want to actively "spy" on users, for ethical and RGPD reasons. Even if we don't store user information, we are not sure of the legal implication on such data collection, so we preferred to avoid it.

Lastly, there are technical reasons. The twitter API is quite limiting when it comes to link mining. So, looking at users' friend network would quickly exhaust our allowed API calls.

Yet, we agree, one should be able to get better results by gathering network information, that's why we did explore this path, but we let it out to future works for these particular reasons.

5. Low Recall and F-score are not explained (I think the reviewers had already mentioned that). Either explain why this result is sufficient and cannot be higher (is it so for tweets because of their length?) or at least highlight the results that you orient to and explain why recall and F-score are not that important (which I honestly doubt).

Reply: You are right, we should have discussed the F-1 score for the NER task. We did not in the previous version because we consider that it is not a low score but rather a good score. NER in tweets is a very complex task. If we look at current benchmarks for NET in tweets (e.g. https://paperswithcode.com/dataset/wnut-2016-ner), the recall and F1 scores are even lower due to the properties of tweets: they are short and often written with shorthands and abbreviations. For instance, in our dataset, we find a tweet that can be translated as "the ground shakes in stras" where "stras" means Strasbourg, the city in the east of France. Those instances are very complex to recognize. We added a similar discussion in the text to explain those values.

Reviewer #4: This manuscript presents an entity-linking pipeline for geolocating French Tweets posted during natural disasters. Furthermore, the manuscript introduces an annotated entity-linking data set consisting of French Wikipedia corpus and French tweets collected during major disasters. I would recommend accepting this paper after clarifying a few points (elaborated below).

Strengths:
• The qualitative evaluation is good, demonstrating the utility of the proposed model for disaster response.
• The paper is well-written. The organization of the paper could be improved, but it remains easy to follow.
• The creation of an annotated data set that can be used for disaster response Named Entity Recognition and Entity Linking tasks in other languages is a valuable contribution, given that the mainstream research in this area is focusing on English data.

Concerns:
• The authors should clarify whether the proposed entity-linking pipeline presented in this manuscript presents a novel contribution. Are there similar entity-linking models that have been proposed to address this specific issue? Is the proposed architecture unique? A comparison with previous works is recommended.

Reply: The novelty of this approach comes primarily from (1) the use of entity-linking to perform geolocation and (2) the updates made on the bi-encoder to simultaneously retrieve and classify all

mentions in a text. It does not really change the way people do entity-linking, as the principles remain the same (retrieve then rerank), but it does improve the efficiency of exisisting systems by a lot.
We made these contribution clearer both in the introduction and Section 3.

There are other minor novelties, such as the training set used to train our cross-encoder.

• The cross-encoder component in the proposed pipeline is not clear. Clarifications are needed on whether this cross-encoder was trained separately or on the output of the bi-encoder. It will be helpful to add a figure illustrating all the pipeline's components and how they are linked to each other's.

Reply: All models are trained separately. However, the cross-encoder depend on the bi-encoder, as it is trained on data generated by the bi-encoder. We added more details in the paper, but the general idea is that we need to train the cross-encoder on *realistic* data. During inference, the cross-encoder will be asked to rerank candidates retrieved by the bi-encoder, but they will (should) be very similar to each others. Reranking similar objects is a very difficult task, so we need to introduce this complexity during the training of the model. That's why we cannot just randomly sample negative example to train the model. We need to *actively mine* them. We can do this manually, but it's easier and more maintainable to generate them automatically using the trained bi-encoder.

• The categories of 'RISKNAT' and 'DAMAGES' (page 11) are unclear. Are they location-related categories? How do they differ from the 'GEOLOC' category? The authors provided a few examples of the 'RISKNAT' category in page 17 "information describing the phenomenon" such as "heavy rain" and "strong gusts of wind", which do not seem to be location related. It would be beneficial if the authors explain more what these two categories are referring to in page 11.

Reply: The categories of 'RISKNAT' and 'DAMAGES' clearly differ from the 'GEOLOC' one, as they do not contain any geographic information. These categories are considered here because we consider them important for the construction of a "common operational picture": in particular, their geospatial analysis (via inferred geolocation) seems very relevant, for example to identify highly impacted sectors (e.g. spatial concentration of messages associated with the 'DAMAGES' category) or natural induced effects such as landslides (e.g. spatial concentration of messages associated with the 'RISKNAT' category). The explanation of these two categories in page 11 has been modified to be more explicit.

• In section 5.2 (Results), the authors stated "Recently, a comparison of several NER models showed that state-of-the-art F1 scores are smaller than 75% [64], meaning that the results of the Bi-Encoder are excellent". It is not clear whether the F1 scores mentioned here are those trained and tested on French data. If it is the case, I think the F1 score obtained on French and English data are not directly comparable. There might be some variations in the way locations are mentioned in these two languages. Even though the scope of this paper is French tweets, I would suggest training and testing the NER component of the proposed pipeline on an existing English Benchmark (e.g., the one proposed in "Suwaileh R, Elsayed T, Imran M, Sajjad H. When a disaster happens, we are ready: Location Mention Recognition from crisis tweets. International Journal of Disaster Risk Reduction. 2022; p. 103107") and compare its performance to the state-of-the-art NER models to support the above conclusion.

Reply: You are completely right about the NER scores, they correspond to benchmarks with English tweets, and there is no theoretical guarantee that the task is similarly complex in French. However, we made this assertion based on recent research showing that there is no major difference in the NER task between English and French, and other languages (https://doi.org/10.1016/j.artint.2012.03.006). We added this reference to the manuscript to explain that the NER score in French can be compared to other NER scores in English.

Regarding your suggestion to test on NER benchmarks, such as the ones used in the cited paper, it is indeed a very good idea. However, as explained in our previous round of reviews, the first author has now left our organisations, and he does not have enough time now with his new job to conduct these new experiments. For the other authors, it would be much more time consuming to carry out these experiments. This is why we decided not to follow your suggestion, and we hope you will not hold it against us.
